# Links Between Ethylene and Sulfur Nutrition—A Regulatory Interplay or Just Metabolite Association?

**DOI:** 10.3389/fpls.2015.01053

**Published:** 2015-12-01

**Authors:** Anna Wawrzynska, Grzegorz Moniuszko, Agnieszka Sirko

**Affiliations:** Institute of Biochemistry and Biophysics Polish Academy of SciencesWarsaw, Poland

**Keywords:** abiotic stress, ethylene, sulfur nutrition, LSU, SLIM1, signaling

## Abstract

Multiple reports demonstrate associations between ethylene and sulfur metabolisms, however the details of these links have not yet been fully characterized; the links might be at the metabolic and the regulatory levels. First, sulfur-containing metabolite, methionine, is a precursor of ethylene and is a rate limiting metabolite for ethylene synthesis; the methionine cycle contributes to both sulfur and ethylene metabolism. On the other hand, ethylene is involved in the complex response networks to various stresses and it is known that S deficiency leads to photosynthesis and C metabolism disturbances that might be responsible for oxidative stress. In several plant species, ethylene increases during sulfur starvation and might serve signaling purposes to initiate the process of metabolism reprogramming during adjustment to sulfur deficit. An elevated level of ethylene might result from increased activity of enzymes involved in its synthesis. It has been demonstrated that the alleviation of cadmium stress in plants by application of S seems to be mediated by ethylene formation. On the other hand, the ethylene-insensitive *Nicotiana attenuata* plants are impaired in sulfur uptake, reduction and metabolism, and they invest their already limited S into methionine needed for synthesis of ethylene constitutively emitted in large amounts to the atmosphere. Regulatory links of EIN3 and SLIM1 (both from the same family of transcriptional factors) involved in the regulation of ethylene and sulfur pathway, respectively, is also quite probable as well as the reciprocal modulation of both pathways on the enzyme activity levels.

## Introduction

**Figure 1:**
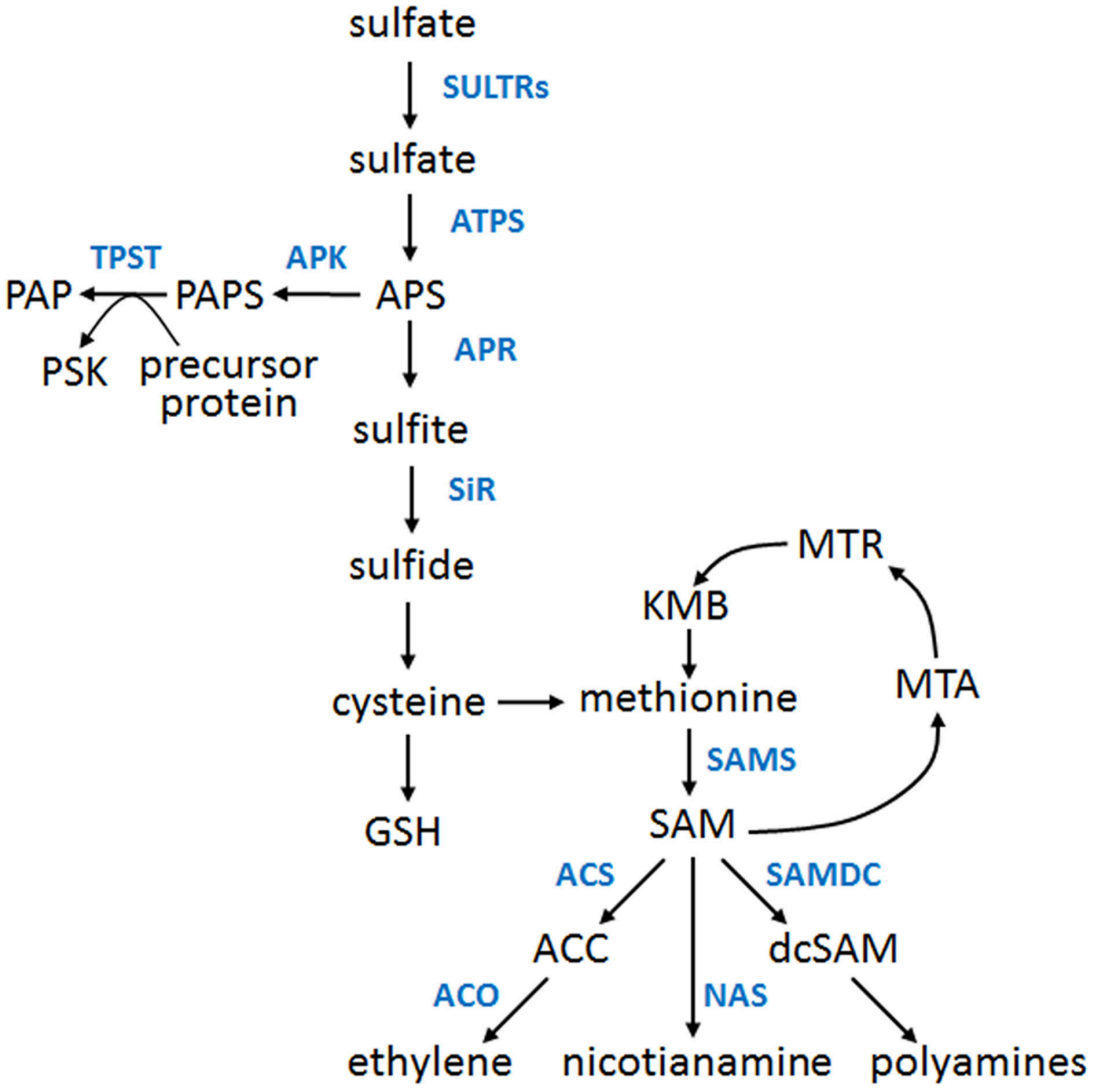
**An overview of the sulfur assimilation pathway and major sulfur metabolites**. Only the selected metabolites (black fonts) and selected enzymes (blue fonts) of the pathway are presented. ACC, 1-aminocyclopropane-1-carboxylate; ACO, ACC oxidase; ACS, ACC synthase; APK, APS kinase; APR, APS reductase; APS, adenosine 5′-phosphosulfate; ATPS, ATP sulfurylase; dcSAM, decarboxylated SAM; GSH, reduced glutathione; KMB, α-keto-γ-methylthiobutyric acid; MTA, S-methyl-5′-thioadenosine; MTR, S-methyl-5-thio-D-ribose; NAS, nicotianamine synthase; PAP, 3′-phosphoadenosine 5′-phosphate; PAPS, 3′-phosphoadenosine 5′-phosphosulfate; PSK, phytosulfokine; SAM, S-adenosylmethionine; SAMDC, SAM decarboxylase; SAMS, SAM synthase; SiR, sulfite reductase; SULTR, sulfate transporter; TPST, tyrosylprotein sulfotransferase.

Sulfur (S) is an important macronutrient for all organisms. Plants can metabolize inorganic sulfur that is taken up from the soil in the oxidized form (sulfate) and then it is reduced and incorporated into a broad range of primary and secondary metabolites. Some of them serve as precursors of other important (but not S-containing) cellular compounds. A schematic overview of the S assimilation pathway, including most of the related metabolites, is shown in Figure 1. The crosstalk between sulfur assimilation and ethylene signaling in plants attracts more attention because of the growing number of data concerning the influence of S nutrition on ethylene signaling and production, as well as the impact of ethylene on the expression of S genes, activity of S enzymes and level of S metabolites (Iqbal et al., 2013). Here, we briefly summarize the most important facts and observations related to the links between ethylene and S nutrition and propose a working model of the complex signaling and regulatory interplay between these two factors.

## Sulfur metabolites as precursors in ethylene synthesis

Methionine (Met), a sulfur-containing amino acid is a substrate for S-adenosylmethionine synthase (SAMS) responsible for the synthesis of S-adenosylmethionine (SAM or AdoMet), an important metabolite in animals and plants (Fontecave et al., 2004; Roje, 2006). SAM serves as a donor of methyl, amino, ribosyl, and aminoalkyl groups. It is also a source of controlled 5′-deoxyadenosine radicals. In plants, SAM is a precursor of polyamines (PA), nicotianamine (NA) used to produce phytosiderophores, and ethylene. Production of ethylene is a two-step reaction with 1-aminocyclopropane-1-carboxylate (ACC), as a product of the first reaction, catalyzed by ACC synthase, and the substrate for the second reaction catalyzed by ACO (ACC oxidase; Figure 1). Met and SAM used for PA, NA and ethylene biosynthesis are recycled in the Met salvage cycle (known also as a Yang cycle). Noteworthy, soluble Met is apparently a rate-limiting metabolite of ethylene biosynthesis (Katz et al., 2006; Bürstenbinder et al., 2007), however for further details on the additional salvage cycles, regulatory circuits and complex relationships between the metabolites and enzymes, please see the reviews (Amir, 2010; Sauter et al., 2013). A new player, a plasma membrane receptor-like kinase, FERONIA, involved in the regulation of SAMS in *Arabidopsis thaliana*, has been recently reported (Mao et al., 2015).

Additional complexity is added by the fact that ACC seems to have more functions than just being the precursor of ethylene. It is a subject of short- and long-distance dedicated transport, can be conjugated to form three different derivatives. It also seems to be a signaling molecule by itself (Van De Poel and Van Der Straeten, 2014).

## Sulfur nutrition affects ethylene synthesis during various stresses

Sulfur nutrition has been reported to modulate the stress response by increasing ethylene production in several stresses. The most intensively studied is cadmium (Cd)-induced stress. The results of experiments with mustard and wheat indicated that the reduced sensitivity of plants to ethylene due to Cd exposure is elevated with additional S supply. S application increased photosynthesis and dry mass, and resulted in the alleviation of oxidative stress by increasing the levels of antioxidant compounds, such as reduced glutathione (GSH; Masood et al., 2012; Asgher et al., 2014; Khan et al., 2015).

Drought stress has been shown to down-regulate S metabolism and ethylene enzymes in medicago roots and nodules (Larrainzar et al., 2014). Also in cassava grown during the dry season, the association of ethylene level with sulfur metabolism and GSH level in root cortex tissues was observed (Saithong et al., 2015). The regulatory aspect of various primary and secondary S metabolites in relation to drought response, including the role of 3′-phosphoadenosine 5′-phosphate (PAP) produced from 3′-phosphoadenosine 5′-phosphosulfate (PAPS) in retrograde signaling, were recently reviewed (Chan et al., 2013). Besides, the authors underline that various osmoprotectants (for example PA), accumulating during drought stress, require restoring the sulfur moiety in SAM through the Yang cycle.

Moreover, it has been shown that the effects of salt stress (inhibition of photosynthesis) in mustard can be reversed by excess S, and this reversal involved ethylene because the inhibition of ethylene biosynthesis counteracted the effects of S excess on salt stress alleviation (Nazar et al., 2014). The authors suggest that under salt stress, S was used for GSH synthesis instead of ethylene formation, while excess S resulted in increased ethylene, stimulating more efficient utilization of intracellular CO_2_ for photosynthesis (Nazar et al., 2014).

Several clusters of genes upregulated by iron (Fe) deficiency in Arabidopsis were reported; one of them contains genes with a function predominantly linked to S assimilation and genes induced by S deficiency (Ivanov et al., 2012). However, the regulatory links between Fe and S metabolism are still unclear. There are contradicting reports on the influence of Fe nutrition on the expression of genes encoding sulfate transporters. On one hand, genes encoding two high affinity sulfate transporters were induced during Fe starvation in tomatos (Paolacci et al., 2014), while, on the other hand, Fe starvation reduced the expression of *SULTR1;1*, encoding the high affinity sulfate transporter in the Arabidopsis roots (Forieri et al., 2013). Moreover, S deprivation limited Fe-deficiency responses in tomatos (Zuchi et al., 2009), however additional S nutrition ameliorated the damages in photosynthetic apparatus caused by Fe deficiency in oilseed rape (Muneer et al., 2014). The existence of co-regulation of S and Fe metabolism was recently discussed in terms of a possible role of several metabolic processes, including the involvement of [Fe–S] clusters in creating the important feedback signal leading to adjustment of the metabolism, for example, Fe and S uptake (Forieri et al., 2013). The role of ethylene in such co-regulation is unclear.

Transcriptomic analysis of grape berries treated with SO_2_ revealed the reprogramming of transcriptome after treatment. Transcripts involved in auxin, ethylene and jasmonate signaling were strongly upregulated, including transcripts encoding auxin responsive proteins, ACC synthase, ACC oxidase, ethylene responsive proteins and lipoxygenase (Giraud et al., 2012). In addition to the S supply, S limitation also results in induction of the ethylene pathway. For example, a short-term S limitation (2 days) resulted in increased expression of some ethylene-related genes (Lewandowska et al., 2010) and elevated ethylene level (Moniuszko et al., 2013) in tobacco and a long-term S limitation (35 days) resulted in an increased amount of ACS in oilseed rape plants (D'hooghe et al., 2013). Interestingly, no increase of ethylene synthesis was observed when tomato plants were starved for S and Fe simultaneously (Zuchi et al., 2009).

## Vice versa: ethylene and ethylene signaling affects sulfur metabolism

Accumulation of APR activity as a result of the treatment of Arabidopsis with 0.2 mM ACC has been shown (Koprivova et al., 2008). Additionally, ethylene has been shown to increase ATP-sulfurylase activity and S accumulation in mustard (Iqbal et al., 2012). However, these few reports cannot be extrapolated into a universal hypothesis that ethylene stimulates S metabolism and accumulation. In fact, despite the above-mentioned increased production of ethylene during a response to S deficiency in *Nicotiana tabacum* (Moniuszko et al., 2013) and *Solanum lycopersicum* (Zuchi et al., 2009), the transcription of only a fraction of ethylene responsive genes was affected. Similar results could be extracted from microarray studies on Arabidopsis (Hirai et al., 2003; Nikiforova et al., 2003).

Consistent lack of correlation of the transcriptomics data with ethylene measurements suggests an association of S deficit with ethylene signaling machinery rather than with ethylene production. Moreover, recent reports put forward the possible occurrence of the cross talk between Sulfur LIMitation 1 transcription factor (SLIM1, described in the next chapter) and ethylene receptors. The re-analysis of the Arabidopsis microarray data showed that silver nitrate mimics the signal for perception of sulfur deficiency in plants at the transcriptome level (Moniuszko, 2015). The author identified 20 genes that were similarly regulated under S deficit and AgNO_3_ treatment. Noteworthy, all 20 are considered S deficiency markers, and three of them (*LSU1, LSU2*, and *SULTR1;2*) are candidates for regulators of responses to S deficiency (Moniuszko et al., 2013; Zhang et al., 2014). Only two of them (APR2 and APR3) cannot be linked with SLIM1 during the plant's early response to S deficiency. The analysis also showed that the similarity between S deficit and AgNO_3_ treatment is rather linked to the silver nitrate action on ethylene receptors than to other AgNO_3_ effects (Moniuszko, 2015).

This mostly theory driven conclusion is supported by previously overlooked studies. It has been shown that *Eruca sativa* proteomic response to Ag+ ions is related to S metabolism (Vannini et al., 2013). The observed changes in S metabolites of *E. sativa* due two Ag+ exposures strongly suggest SLIM1 involvement. In addition, the heterologous expression of the Arabidopsis ethylene receptor gene, *etr1-1* (which encodes mutated ETR1 protein unable to relay ethylene signal after hormone binding), in *N. attenuata* resulted in impaired sulfate uptake and S metabolism (Meldau et al., 2013). Abnormal phenotypes of such seedlings under optimal sulfate supply (similar to plants grown under S deficit) suggest a defect in SLIM1 action as a result of changes in ethylene signaling at the receptor level. Apparently, the etr1-1 receptor, despite (and in addition to) its inability to properly function in a classic linear ethylene-signaling pathway, was mimicking the signal of S deficiency.

On the other hand, proper ethylene signaling was found to be necessary for increased GSH accumulation after ozone treatment. In the *ein2* Arabidopsis mutant plants, 6 h after ozone exposure, the increment of GSH level was much lower than in the control plants (Yoshida et al., 2009). Research involving the extrapolation of such regulation on different stresses falls way behind. Presently, the cross talk between GSH biosynthesis and ethylene signaling has been proposed only for Cd and drought (Masood et al., 2012; Saithong et al., 2015). Both cases have been discussed above regarding the S nutrition effect on ethylene production. However, we want to emphasize here that in the case of Cd treated mustard, the effects of additional S supply were reversed by the ethylene biosynthesis inhibitor, aminoethoxyvinylglycine (AVG), and that similar effects were achieved by additional S supply and ethephon treatment (Masood et al., 2012). Thus, the authors suggested a prominent role of ethylene (possibly on GSH biosynthesis) in S-induced alleviation of Cd stress. However, this might be the reflection of a switch between the ethylene receptors' role in S status sensing and linear ethylene signaling, as discussed in a recently proposed model (Moniuszko, 2015). Nevertheless, further studies are needed to clarify the exact molecular mechanism behind the observed effects of ethylene and ethylene signaling on sulfur metabolism and its regulation.

## Possible regulatory mechanisms responsible for coupling sulfur and ethylene signaling and metabolism

The transcriptional control of gene expression very often serves to reprogram plant metabolism in order to cope with environmental challenges. So far the only described transcription factor exclusively assigned to affect gene expression during S deficiency is SLIM1 from Arabidopsis (Maruyama-Nakashita et al., 2006). Certainly, attracting attention in the perspective of this review is the fact that SLIM1 belongs to the same plant protein family as EIN3, the main transcription factor controlling the expression of ethylene-responsive genes. It was initially identified as *ETHYLENE-INSENSITIVE-LIKE 3 (EIL3)* coding for a putative transcription factor of unknown function (Guo and Ecker, 2004). Analyses of the knockout mutants revealed that SLIM1 affects the expression of various genes facilitating the increased flux through the sulfate assimilation pathway and translocation of sulfate to the shoot, but it also controls the degradation of glucosinolates under sulfur deficient conditions (Maruyama-Nakashita et al., 2006). The functional complementation of the *slim1* mutant was only successful with SLIM1 and not any other protein member of EIL family, pointing out its specificity. Moreover, the treatment of plants with the precursor of ethylene, ACC, does not affect the transcription of any of SLIM1-dependent genes (Maruyama-Nakashita et al., 2006). It is tempting to speculate that the C-terminal part of the EIL proteins is responsible for that functional separation since all of them are highly homologous to one another, mainly in their N-terminal half of around 300 amino acid residues. All six members of the Arabidopsis EIL family share highly acidic N-terminal amino acids, five small clusters of basic amino acids scattered mostly in the first half of the protein and a proline-rich domain (Chao et al., 1997). SLIM1 served as a template to model the unique DNA-binding domain of the EIL family, consisting of five alpha helices, packed together into a globular shape as a whole (Yamasaki et al., 2005). The DNA-binding abilities of EIN3, EIL1, and EIL2 proteins have been demonstrated with ethylene response DNA elements, which are 28-nt imperfect palindromes, using an electro-mobility shift assay (Solano et al., 1998). The interaction of SLIM1 with those sequences is very unstable and is only detectable with surface plasmon resonance (Yamasaki et al., 2005), demonstrating the binding preferences between EIL family members. SLIM1 strongly binds to 20-nt consensus, called the UPE-box, which is only present in the promoters of eight genes that are strongly induced by S deficiency in Arabidospis (Wawrzynska et al., 2010). Yet three of these genes encode proteins from the LSU family, homologs of tobacco UP9C protein (Sirko et al., 2014). Silencing of *UP9C* expression in tobacco led to disturbances of the ethylene signaling and synthesis pathways during conditions of S deficiency (Moniuszko et al., 2013).

In contrast to EIN3, not much is known about SLIM1 posttranslational modifications or its interaction with other proteins (Wawrzynska and Sirko, 2014). Its transcription level is not modulated by the changes of S conditions (Maruyama-Nakashita et al., 2006); however a strong elevation is observed in root tissue during Fe deficiency (Garcia et al., 2010). SLIM1 can bind with MYB72, which together with MYB10 induce the nicotianamine synthase gene *NAS4* governing proper homeostasis of Fe during its deficiency. However, this also triggers jasmonate/ethylene-dependent systemic resistance (Van Der Ent et al., 2008; Palmer et al., 2013). On the other hand, MYB72 is a direct target of FIT, a central regulator of Fe assimilation in roots (Sivitz et al., 2012). FIT abundance is controlled by interaction with EIN3, which reduces FIT proteasomal degradation leading to a higher level of expression of the genes involved in Fe acquisition (Lingam et al., 2011). Both SLIM1 and EIN3, therefore, seem to tune up Fe homeostasis when plants meet the conditions of deficiency.

Despite the possible cross talk between ethylene and S deficiency signals on the level of EIN3 and SLIM1 transcriptional factors, the regulation on the level of stability of enzymes involved in ethylene synthesis might be also envisaged. Such possibilities might be deduced from the reported interaction of the above-mentioned UP9C protein with ACO in tobacco (Moniuszko et al., 2013). Interestingly, many members of the LSU family are induced during S starvation and it is tempting to speculate that the interaction of these proteins with ACO serves some regulatory reason because of the lack of S-deficiency induced elevation of ethylene level in tobacco plants with lowered expression of *UP9C* (Moniuszko et al., 2013). Notably, the posttranslational regulation of ACS is a well-known phenomenon; however information about such regulation of ACO is thus far limited. Nevertheless, this possibility is supported by the transcriptomic-based kinetic model for ethylene synthesis in tomato fruits that indicates the existence of potential posttrancriptional regulation of ACO (Van De Poel et al., 2014).

Moreover, the small (five amino acids) peptide, phytosulfokine (PSK), a growth factor containing sulfated tyrosine might be an additional player in this complex signaling and regulatory network. PSK is produced from an 80-amino-acid-long precursor (there exist six *PSK* genes in Arabidopsis) via tyrosine sulfation and proteolytic processing (Matsubayashi, 2014; Sauter, 2015). Recent analysis of the Arabidopsis *tpst-2* mutant defective in tyrosylprotein sulfotransferase revealed that PSK suppresses ethylene production (Wu et al., 2015).

**Figure 2:**
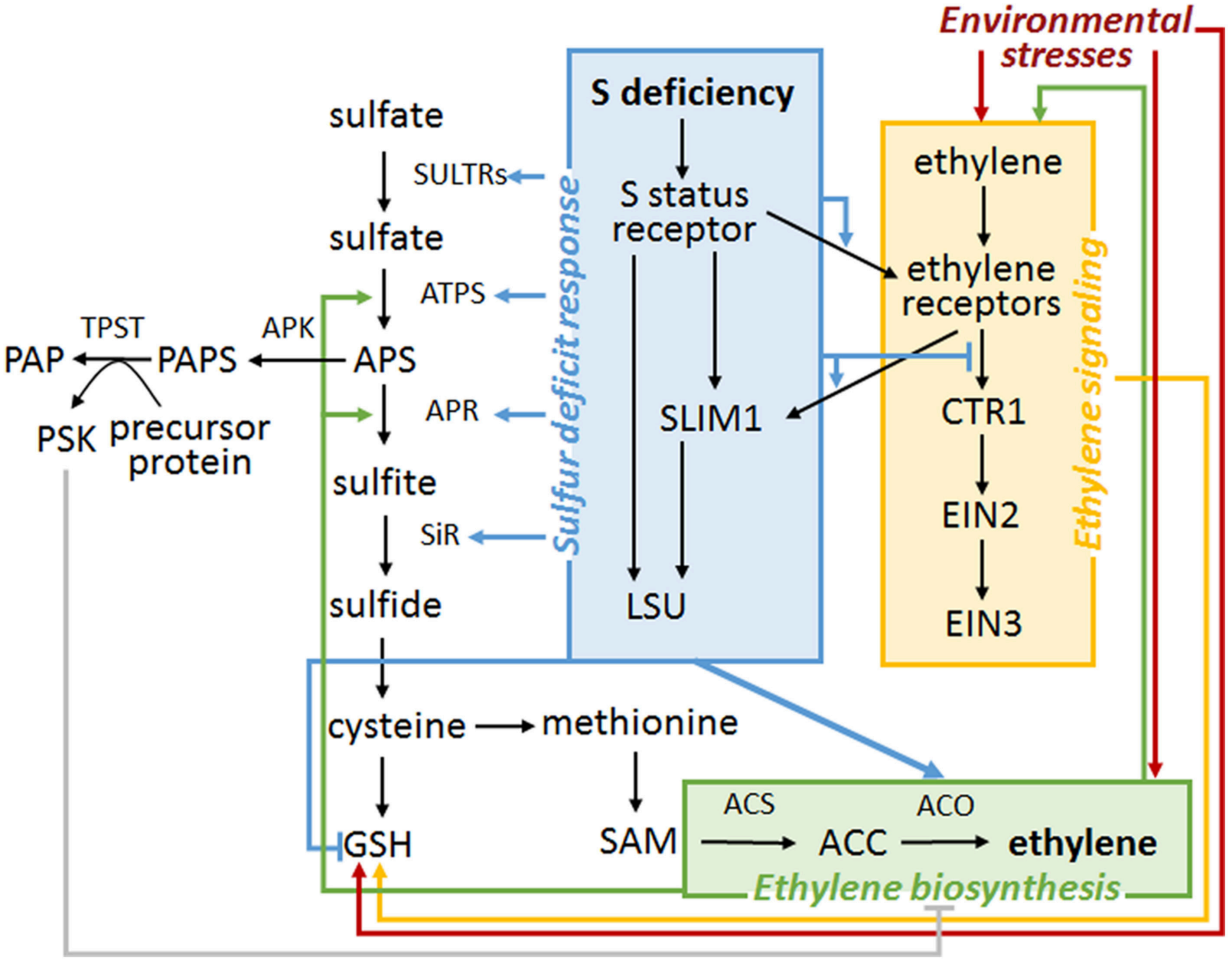
**A hypothetical model of regulatory links between S− and ethylene sensing and signaling**. Only the selected metabolites, enzymes and other players are presented. The black arrow represents one-step or multiple-step signaling or metabolic pathway progress. Colored arrows (gray, red, blue, green, orange) represent regulatory mechanisms reported in the published studies. At the current stage, most of these mechanisms are obscurely documented and need further research. Additionally, the S status sensor is elusive.

The hypothetical model explaining possible co-regulation of sulfur and ethylene signaling in plants is shown in Figure 2. According to the model, S availability modulates ethylene sensitivity due to a switch of ethylene receptor function. S deficiency might also affect ethylene production by stabilizing ACO level or activity. On the other hand, ethylene production is negatively affected by the sulfated phytohormone, PSK. The functional ethylene pathway is necessary for increased level of GSH in some stresses. Moreover, ethylene seems to stimulate the activity of several enzymes involved in S assimilation.

## Conclusions

Ethylene production and sulfur assimilation pathways have close boundaries and share some metabolites. Thus, they might have also common regulatory elements. Although numerous observations suggest that these two pathways might indeed share some sensing or signaling elements, the molecular details are still obscure. Additional experiments are required to clarify and explain some contradicting and imprecise data. Answers to the following questions might help to elucidate the molecular basis of the postulated cross-talk of both signaling pathways: What is the S deficiency signal? What molecules function as the S status receptors? What factors are directly involved in linking these two pathways?

## Author contributions

AS drafted the manuscript. All authors were involved in the writing process and preparing the final version.

## Funding

This work was supported by Narodowe Centrum Nauki (National Science Center), Grants 2014/15/B/NZ3/04854 and 2014/15/B/NZ1/01887.

### Conflict of interest statement

The authors declare that the research was conducted in the absence of any commercial or financial relationships that could be construed as a potential conflict of interest.
